# Propolis Reduces Phosphatidylcholine-Specific Phospholipase C Activity and Increases Annexin a7 Level in Oxidized-LDL-Stimulated Human Umbilical Vein Endothelial Cells

**DOI:** 10.1155/2014/465383

**Published:** 2014-04-22

**Authors:** Hongzhuan Xuan, Zhen Li, Jiying Wang, Kai Wang, Chongluo Fu, Jianlong Yuan, Fuliang Hu

**Affiliations:** ^1^School of Life Science, Liaocheng University, Liaocheng 252059, China; ^2^College of Animal Sciences, Zhejiang University, Hangzhou 310029, China; ^3^Institute of Animal Science and Veterinary Medicine, Shandong Academy of Agricultural Sciences, Jinan 250100, China

## Abstract

To understand the mechanisms underlying the regulating dyslipidemia action of Chinese propolis and Brazilian green propolis, we investigated their effects on phosphatidylcholine-specific phospholipase C (PC-PLC) activity and annexin a7 (ANXA7) level which play crucial roles in the control of the progress of atherosclerosis. Furthermore, active oxygen species (ROS) levels, nuclear factor-KappaB p65 (NF-**κ**B p65), and mitochondrial membrane potential (MMP) were also investigated in oxidized-LDL- (ox-LDL-) stimulated human umbilical vein endothelial cells (HUVECs). Our data indicated that the treatment of both types of propolis 12.5 **μ**g/mL significantly increased cell viability and attenuated apoptosis rate, increased ANXA7 level, and decreased PC-PLC activity. Both types of propolis also inhibited ROS generation as well as the subsequent MMP collapse, and NF-**κ**B p65 activation induced by ox-LDL in HUVECs. Our results also indicated that Chinese propolis and Brazilian green propolis had similar biological activities and prevented ox-LDL induced cellular dysfunction in HUVECs.

## 1. Introduction

Propolis is a brownish resinous material collected by worker bees from the leaf buds of numerous plants like birch, poplar,* Baccharis dracunculifolia*, and* Dalbergia ecastaphyllum* [1–4]. It has been extensively used as a folk medicine since ancient time because of its special chemical components, strong pharmacological and biological properties, and low toxicity [5].

In recent years, the regulation of dyslipidemia actions of propolis has been widely documented, resulting in the genesis and progression of atherosclerosis. A recent report indicated ethanolic extract of propolis inhibited atherosclerosis in ApoE-knockout mice [6]. Furthermore, we also reported that Chinese propolis regulated lipid metabolism of diabetes* in vivo *by regulating triglycerides, total cholesterol, high-density lipoprotein, and low-density lipoprotein cholesterol [7, 8]. However, the molecular mechanisms underlying such protect effects of propolis have not been fully elucidated.

Phosphatidylcholine-specific phospholipase C (PC-PLC), an important member of phospholipase C family, has been implicated in several cellular signaling pathways such as cell growth, differentiation, senescence, apoptosis, and autophagy of mammalian cells [9–13]. Accumulating evidence demonstrated that PC-PLC was a key inducing element of atherosclerosis and contributed to the progression of atherosclerosis [14]. Pharmacological blockade of PC-PLC inhibited the progression of atherosclerosis [15]. And a recent study indicated that annexin a7 (ANXA7), a member of the annexin family of calcium-dependent phospholipid binding proteins, was an endogenous regulator of PC-PLC. ANXA7 also participated in the progression of atherosclerosis and targeting ANXA7 inhibited atherosclerosis in apoE^−/−^ mice. ANXA7/PC-PLC signaling pathway may represent a novel target for the treatment of atherosclerosis [16].

Chinese propolis affected PC-PLC activity. Our previous study indicated that Chinese propolis played an anti-inflammatory role partly through its inhibitory effect on the activity of PC-PLC [17]. Considering the important roles of ANXA7 and PC-PLC in atherosclerosis and propolis modulated atherosclerosis and affected PC-PLC activity, we hypothesized that propolis may also affect ANXA7, the endogenous regulator of PC-PLC. In present study we tested the hypothesis by investigating the effects of Chinese propolis and Brazilian green propolis on PC-PLC activity and ANXA7 level in ox-LDL-stimulated HUVECs; ox-LDL plays crucial role in triggering the development of atherosclerosis. Furthermore, we investigated the effects of both types of propolis on reactive oxygen species (ROS) levels, nuclear factor-KappaB p65 (NF-*κ*B p65), and mitochondrial membrane potential (MMP) which were regulated by PC-PLC in HUVECs.

## 2. Materials and Methods

### 2.1. Chemicals and Reagents

DMEM was from Gibco (USA). Fetal bovine serum (FBS) was from Hyclone Lab Inc. (USA). L-*α*-phosphatidylcholine, 3-(4,5-dimethylthiazol-2-yl)-2,5-diphenyltetrazolium bromide (MTT), 2′,7′-dichlorodihydrofluorescein diacetate (DCFH), and JC-1 were from Sigma Co. (USA). Acridine orange was from Amresco (USA). Ox-LDL was from Beijing Union-Biology Co. (China). Primary antibodies against ANXA7, NF-*κ*B p65, GAPDH, and secondary antibody (horseradish peroxidase) were from Santa Cruz Biotechnology (USA). Secondary antibody for immunofluorescence, donkey anti-rabbit IgG Alexa Fluor-488, was purchased from Life Technologies (USA). All other reagents were of ultrapure grade.

### 2.2. Preparation of Propolis Extracts

Propolis used in present study was Chinese propolis and Brazilian green propolis. Both types of propolis had been used in previous studies [17, 18]. The extraction method was as before. Briefly, extracted Chinese propolis was obtained from colonies of honeybees,* A. mellifera* L., in Shandong Province of North China and the main plant origin was poplar (*Populus* sp.). Extracted Brazilian green propolis was collected in Minas Gerais State of Brazil, where* Baccharis dracunculifolia* DC. is the main botanical source. Both types of propolis were stored at −20°C until used. Chinese propolis and Brazilian green propolis samples were extracted with ethanol and then filtered under reduced pressure, and the filter liquid was concentrated under reduced pressure at 40°C until reaching a constant weight and then redissolved in ethanol. The ethanol-extracted Chinese propolis (EECP) and ethanol-extracted Brazilian green propolis (EEBP) had a brown color. The prepared EECP and EEBP were stored under a dry condition at 4°C.

### 2.3. Cell Culture

HUVECs were gifted by Atherosclerosis Research Institute of Taishan Medical University of China purchased from ATCC. HUVECs were cultured in DMEM (high glucose) supplemented with 100 U/mL of penicillin, 100 *μ*g/mL streptomycin, and 10% heat-inactivated FBS at 37°C under humidified 95%–5% (v/v) air and CO_2_.

### 2.4. Exposure of HUVECs to EECP and EEBP

When the HUVEC cultures reached 80% confluence, then the cells were divided for treatment: (a) culture in 3.5% DMEM medium with ethanol at <0.1% (v/v) (control); (b) culture in basal DMEM medium with 45 *μ*g/mL ox-LDL with ethanol at <0.1% (v/v); and (c) culture in basal DMEM medium with 45 *μ*g/mL ox-LDL with EECP and EEBP (12.5 *μ*g/mL), respectively. EECP and EEBP were dissolved in ethanol, with final concentration of ethanol in the culture medium <0.1% (v/v). Ethanol at 0.1% (v/v) did not affect cell viability.

### 2.5. Cell Viability Assay

The viability of HUVECs was determined by MTT assay. HUVECs were seeded in 96-well cell culture plates and grown to 80% confluence and then treated with ox-LDL or EECP and EEBP for 12 and 24 h, respectively. MTT solution was added to each well and incubated for 4 h. The MTT-formazan product dissolved in DMSO to estimate by measuring absorbance at 570 nm in an ELISA plate reader. The viability (%) was expressed as (OD of treated group/OD of ox-LDL group) × 100%. The viability of the ox-LDL group was set at 100%.

### 2.6. Nuclear Fragmentation Assay

The morphological changes of nuclei were detected by acridine orange staining. At 24 h, cells were stained with 5 *μ*g/mL acridine orange at room temperature for 5 min and observed under a laser scanning confocal microscopy (Olympus FV1200, Japan).

### 2.7. PC-PLC Activity Assay

PC-PLC activity assay was performed as the described methods in [19, 20]. Briefly, we used L-*α*-phosphatidylcholine as the substrate of PC-PLC. The optical density was measured at 660 nm. Enzyme activity was expressed as nanomoles per minute per milligram protein.

### 2.8. Immunofluorescence Microscopy

After treatment, cells were fixed with 4% paraformaldehyde and blocked with 5% normal donkey serum for 20 min at room temperature. Cells were incubated with ANXA7 and NF-*κ*B p65 primary antibodies (1/100) at 4°C overnight. After three rinses in 0.1 M phosphate-buffered saline, cells were treated with a corresponding FITC-conjugated secondary antibody (1/200) in a humid chamber at 37°C for 1 h. Cells were rinsed three times with 0.1 M phosphate-buffered saline to eliminate the uncombined secondary antibody. A laser scanning confocal microscope (Olympus FV1200, Japan) was used for fluorescence detection. Analysis used the Image-Pro Plus software (USA). Images are representative of three independent experiments.

### 2.9. Western Blot Analysis

Western blot assay of ANXA7 level was performed as previously described [21]. Thirty micrograms of protein was separated by 12% SDS-PAGE and transferred onto PVDF membrane. The relative quantities of the proteins were evaluated by the use of Quantity One software.

### 2.10. Measurement of ROS Production

ROS production in HUVECs was determined by the use of a fluorescent probe, DCFH, which can be oxidized into fluorescent dichlorofluorescin (DCF) by intracellular ROS [22]. After treating cells for 24 h, cells were incubated with DCFH for 30 min at 37°C. Then cells were washed with basal DMEM medium 3 times and observed on laser scanning confocal microscopy (Olympus FV1200, Japan). The level of ROS was quantified by Image-Pro Plus software (USA). Results were shown as relative fluorescence intensity of three independent experiments.

### 2.11. Measurement of Mitochondrial Membrane Potential

The fluorescent dye JC-1 was used to measure mitochondrial membrane potential. JC-1 exists as a monomer at low mitochondrial membrane potential and emits green fluorescence but forms aggregates and emits red fluorescence at high mitochondrial membrane potential [23]. After treating cells for 24 h, cells were incubated with JC-1 for 15 min at 37°C. Then cells were washed with basal DMEM medium 3 times and observed on laser scanning confocal microscopy (Olympus FV1200, Japan). The mitochondrial membrane potential was quantified by the use of the Image-Pro Plus software (USA). Results were shown as ratio of red to green fluorescence of three independent experiments.

### 2.12. Statistical Analysis

All experiments were performed in duplicate and repeated at least 3 times. Data are expressed as means ± SEM. Statistical analyses were performed using independent *t*-tests and analysis of variance (ANOVA), followed by the Tukey* post hoc test*. A *P* < 0.05 was considered significant.

## 3. Results

### 3.1. Effect of EECP and EEBP on HUVEC Viability

**Figure 1:**
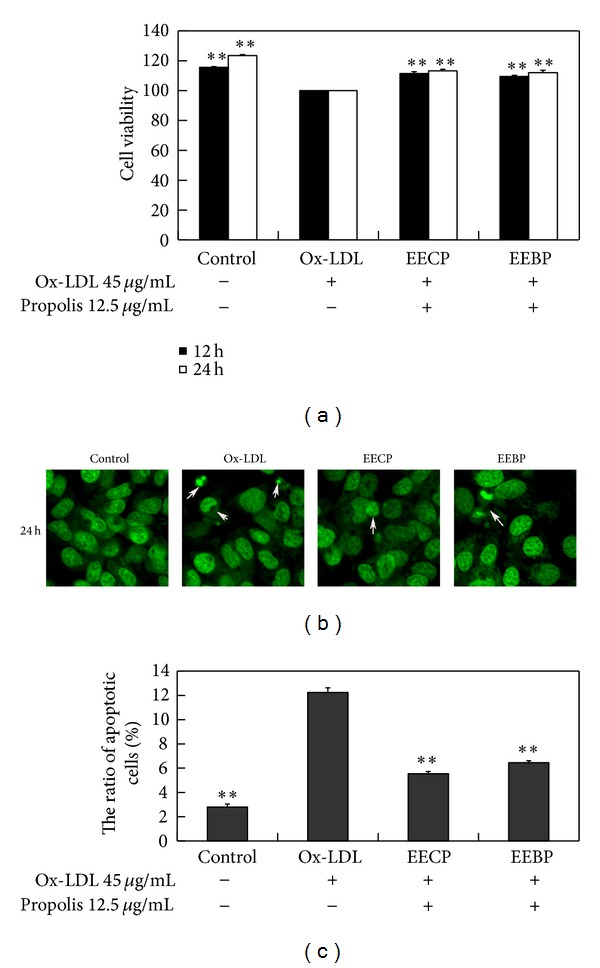
EECP and EEBP increased cell viability and inhibited apoptosis rate in ox-LDL-stimulated HUVECs. (a) Effect of EECP and EEBP on cell viability induced by ox-LDL. EECP and EEBP (12.5 *μ*g/mL) and ox-LDL (45 *μ*g/mL for HUVECs) were used. (b) Effects of EECP and EEBP on nuclear fragment were detected by acridine orange staining. (c) The ratio of apoptotic cells induced by ox-LDL (**P < 0.01 versus ox-LDL group, *n* = 3). Data are means ± SEM.

Ox-LDL is a major cause of endothelial dysfunction. MTT assay revealed that ox-LDL significantly inhibited cell viability, and after treatment with EECP and EEBP 12.5 *μ*g/mL for 12 and 24 h, the cell viability obviously increased compared with ox-LDL group, respectively (**P < 0.01; Figure 1(a)).

### 3.2. Effect of EECP and EEBP on HUVEC Apoptosis

We further examined the effects of EECP and EEBP on HUVEC apoptosis induced by ox-LDL. The results of AO staining showed that there were evidently condensation and fragmentation of chromosomes in ox-LDL group (Figure 1(b)), and cell apoptosis was significantly decreased by EECP and EEBP at 24 h (**P < 0.01; Figure 1(c)).

### 3.3. Effect of EECP and EEBP on PC-PLC Activity

**Figure 2:**
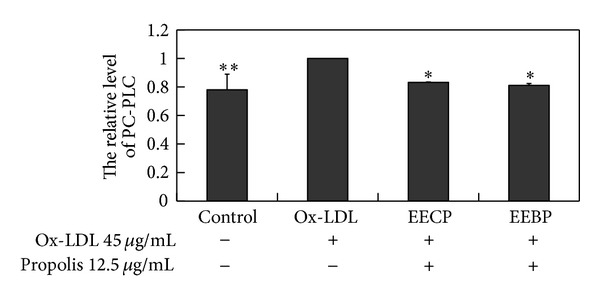
EECP and EEBP decreased PC-PLC activity in ox-LDL-stimulated HUVECs. Cells were treated with EECP and EEBP 12.5 *μ*g/mL for 24 h, respectively (*P < 0.05, **P < 0.01 versus ox-LDL group, *n* = 3).

The activity of PC-PLC in ox-LDL treated HUVECs was significantly increased, whereas EECP and EEBP evidently depressed PC-PLC activity at 24 h (*P < 0.05, **P < 0.01; Figure 2).

### 3.4. Effect of EECP and EEBP on ANXA7 Level

**Figure 3:**
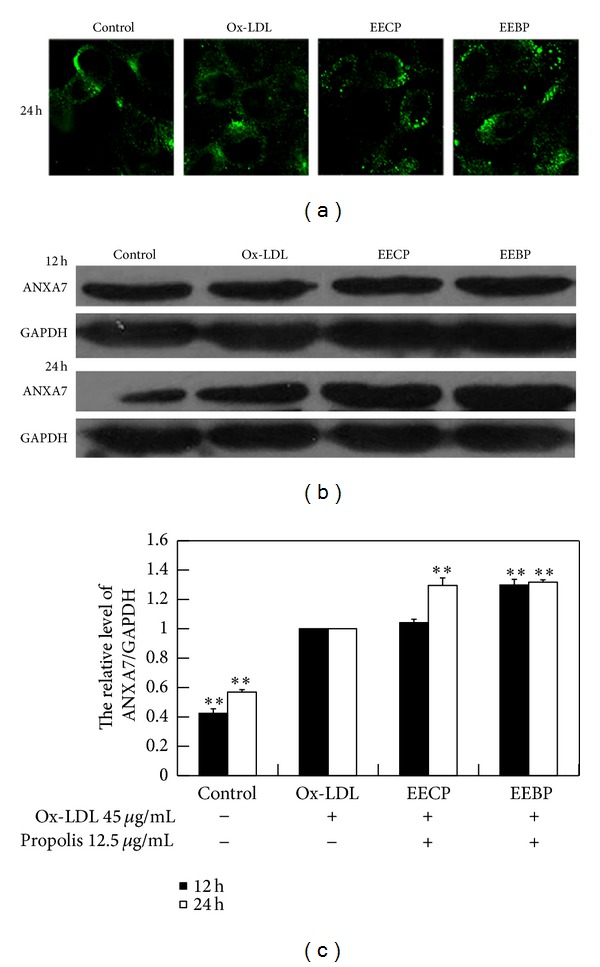
EECP and EEBP increased ANXA7 level in ox-LDL-stimulated HUVECs. (a) Fluorescent micrographs obtained at 24 h. (b) ANXA7 levels were detected by western blot analysis at 12 and 24 h. (c) The hemiquantification of ANXA7 level in HUVECs (**P < 0.01 versus ox-LDL group, *n* = 3).

ANXA7 was the endogenous regulator of PC-PLC. To further investigate the relationship between PC-PLC and ANXA7, we investigated the effect of EECP and EEBP on ANXA7 expression and distribution in cells treated with ox-LDL. Western blotting results showed that EEBP obviously increased ANXA7 level at 12 h, and both EECP and EEBP significantly increased ANXA7 level at 24 h (**P < 0.01; Figure 3). And immunofluorescence assay results showed that cells treated with EECP and EEBP exhibited higher fluorescence intensity of ANXA7 per cell in a noticeable punctate pattern compared with ox-LDL group (Figure 3(a)).

### 3.5. Effect of EECP and EEBP on NF-*κ*B p65 Level

**Figure 4:**
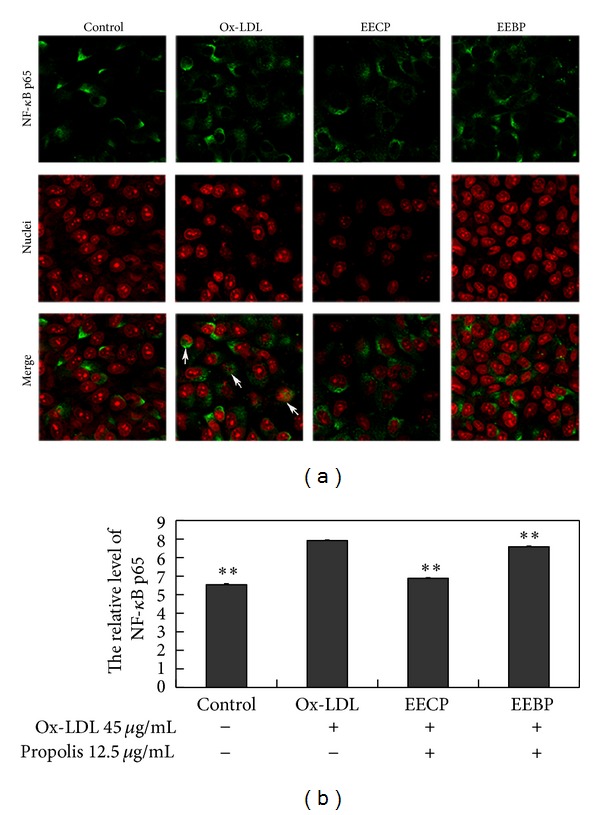
EECP and EEBP decreased NF-*κ*B p65 level and inhibited translocation of NF-*κ*B p65 from cytoplasm to nucleus in ox-LDL-stimulated HUVECs. (a) Fluorescent micrographs obtained at 24 h. Nuclei were counterstained with PI. (b) The relative level of NF-*κ*B p65 in HUVECs (**P < 0.01 versus ox-LDL group, *n* = 3).

Ox-LDL induced NF-*κ*B activation in HUVECs. Both EECP and EEBP 12.5 *μ*g/mL significantly decreased NF-*κ*B p65 level by immunofluorescence assay, and both types of propolis inhibited translocation of NF-*κ*B p65 from cytoplasm to nucleus (**P < 0.01; Figure 4).

### 3.6. Effect of EECP and EEBP on ROS Level

**Figure 5:**
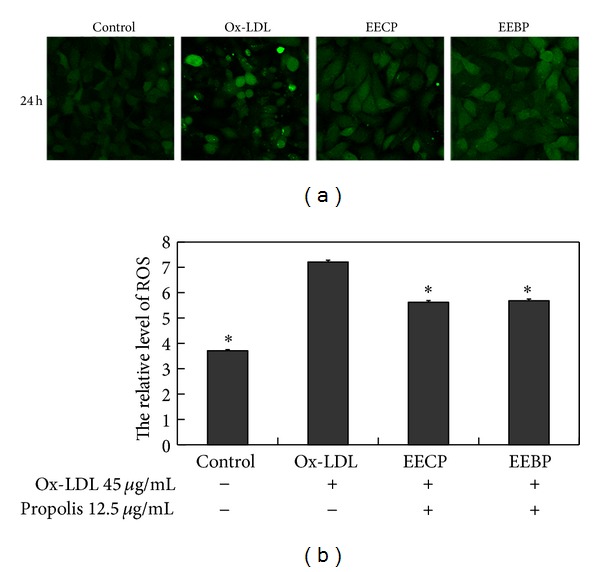
EECP and EEBP decreased ROS level in ox-LDL-stimulated HUVECs. (a) Fluorescent micrographs obtained at 24 h. (b) The relative quantity of ROS level in HUVECs (*P < 0.05 versus ox-LDL group, *n* = 3).

Both EECP and EEBP 12.5 *μ*g/mL significantly decreased ROS generation in HUVECs at 24 h as compared with the ox-LDL group (*P < 0.05; Figure 5).

### 3.7. Effect of EECP and EEBP on Mitochondrial Membrane Potential

**Figure 6:**
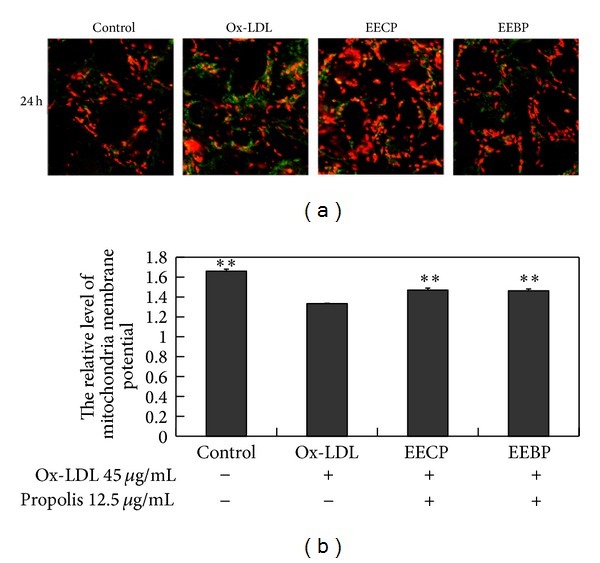
EECP and EEBP increased mitochondria membrane potential in ox-LDL-stimulated HUVECs. (a) Fluorescent micrographs obtained at 24 h. (b) The relative quantity of mitochondrial membrane potential in HUVECs (**P < 0.01 versus ox-LDL group, *n* = 3).

Ox-LDL damages mitochondria membrane potential. Both EECP and EEBP 12.5 *μ*g/mL significantly increased mitochondrial membrane potential compared with ox-LDL group. (**P < 0.01; Figure 6).

## 4. Discussion

Atherosclerosis is considered to be a chronic inflammatory disease. Ox-LDL is believed to be a key step in endothelial cell injury and in the process of initiation and progression of atherosclerotic disease [24, 25]. According to the documents on ox-LDL roles in HUVEC apoptosis [26], in current study, 45 *μ*g/mL of ox-LDL was used. There are more than 300 active components in propolis. Because of different plant source, the chemical constituents of Chinese propolis and Brazilian green propolis are different. Our previous researches suggested that both Chinese propolis and Brazilian green propolis 12.5 *μ*g/mL averted apoptosis and protected HUVECs with nutrient deprivation [17, 18]. Munari et al. (2010) also suggested that 12.5 *μ*g/mL* Baccharis dracunculifolia* extract, the major plant resource of Brazilian green propolis, was the most effective in antigenotoxic chemoprevention [27]. Therefore, we have chosen 12.5 *μ*g/mL Chinese propolis and Brazilian green propolis used in current study and compared their biological activity in ox-LDL-stimulated HUVECs.

Accumulating evidence indicates that PC-PLC plays an important role in progression of atherosclerosis and PC-PLC is an attractive target for antiatherosclerosis therapy [14]. A recent study showed that ANXA7, having different roles in autophagy, tumor suppression, and exocytosis [28–30], was negative regulation of PC-PLC in HUVECs and suggested that ANXA7/PC-PLC signaling pathway may present a novel target to treat atherosclerosis [17]. In present study, the data indicated that both Chinese propolis and Brazilian green propolis reduced PC-PLC activity and increased ANXA7 level in ox-LDL-stimulated endothelial cells, which suggested that ANXA7/PC-PLC might be the targets of both types of propolis in modulating dyslipidemia.

ROS play a critical role in vascular pathology as well as in the maintenance of normal physiological vascular function. Overproduction of ROS will lead to oxidative stress, which cause the endothelial dysfunction and promote the development of many cardiovascular diseases such as atherosclerosis by activating downstream signal molecules such as NF-*κ*B [31]. Our previous study also showed that elevating ROS levels triggered apoptosis in HUVECs with nutrition deprivation [18]. Ox-LDL is a potent inducer of ROS, and this was confirmed in the present study; ROS level was increased in HUVECs treated with ox-LDL, whereas both types of propolis could depress ROS level, which lend support to the theory that ROS scavenging could reduce the risk of cardiovascular diseases [32]. Furthermore, transcription factor NF-*κ*B is activated by high level of ROS [33]. In present study, ox-LDL induced ROS increase and subsequent activation of NF-*κ*B p65 were all attenuated by EECP and EEBP. NF-*κ*B signaling pathway is involved in multiple cell processes including apoptosis, proliferation, and gene expression. Moreover, recent studies have suggested that several natural products including propolis suppress inflammatory responses by regulating the NF-*κ*B pathway [34, 35]. Atherosclerosis is a kind of chronic inflammatory disease. These findings concur with our finding that the transcriptional inhibition of proinflammatory mediators by propolis is associated with the blockade of NF-*κ*B signaling pathway.

Mitochondria are the most important intracellular source of ROS, and elevated ROS levels can also decrease mitochondrial membrane potential [36]. Ox-LDL damages mitochondrial membrane potential, leading the cytochrome c release to induce apoptosis in HUVECs [37]. We previously reported that high concentration of propolis damaged mitochondrial membrane potential in endothelial cells with nutrition deprivation. Here we found that both types of propolis 12.5 *μ*g/mL significantly protected mitochondrial membrane potential. Together with these results, we confirmed the protective effect of propolis on HUVECs induced by ox-LDL, and it may be the major mechanisms of propolis modulating atherosclerosis.

Many studies indicate that propolis from different areas has similar biological activity although the chemical constituents are different. Hu et al. (2011) reported that Chinese propolis and Brazilian green propolis had similar biological activities on streptozotocin-induced type 1 diabetes mellitus in rats [38]. And we also previously found that Chinese propolis and Brazilian green propolis had similar protective effects on hepatocytes injury induced by hydrogen peroxide [39]. In current study, we confirmed that both Chinese propolis and Brazilian green propolis had similar activity on ANXA7 expression and PC-PLC activity and other signal molecules in HUVECs induced by ox-LDL. We proposed that it was not a simple chemical constituent in propolis playing crucial role modulating dyslipidemia diseases; it might be a synergy effect of various chemical constituents of propolis. However, in other cells, such as breast cancer MCF-7 and MDA-MB-231 cells, we found that the cytotoxicity of Chinese propolis and Brazilian green propolis was different. So the activities of propolis on different diseases and cells should be further studied.

Altogether, the present findings indicated that both types of propolis 12.5 *μ*g/mL significantly increased cell viability and attenuated apoptosis rate, increased the expression of ANXA7, and decreased PC-PLC activity. Both kinds of propolis also inhibited ROS generation as well as the subsequent MMP collapse and NF-*κ*B p65 activation induced by ox-LDL in HUVECs, which may be the major mechanisms of propolis protecting endothelial injury and preventing atherosclerosis. Our results also indicated that Chinese propolis and Brazilian green propolis had similar biological activities and prevented ox-LDL induced cellular dysfunction in HUVECs. Both types of propolis may be potent alternative drugs for the prevention of atherosclerosis. However, the mechanism of propolis regulating dyslipidemia should be further studied.
